# Aging‐induced Akt activation involves in aging‐related pathologies and Aβ‐induced toxicity

**DOI:** 10.1111/acel.12989

**Published:** 2019-06-11

**Authors:** Yu‐Ru Chen, Yu‐Hsuan Li, Tsung‐Chi Hsieh, Chih‐Ming Wang, Kuan‐Chung Cheng, Lei Wang, Tzu‐Yu Lin, Chun Hei Antonio Cheung, Chia‐Lin Wu, HsuehCheng Chiang

**Affiliations:** ^1^ Department of Pharmacology National Cheng‐Kung University Tainan Taiwan; ^2^ Institute of Basic Medical Sciences College of Medicine National Cheng Kung University Tainan Taiwan; ^3^ School of Pharmacy College of Medicine National Cheng Kung University Tainan Taiwan; ^4^ College of Life Science and Technology Beijing University of Chemical Technology Beijing China; ^5^ Department of Biochemistry and Graduate Institute of Biomedical Sciences College of Medicine Chang Gung University Taoyuan Taiwan; ^6^ Department of Neurology Chang Gung Memorial Hospital Linkou Taiwan

**Keywords:** γ‐secretase, aging, Akt, Alzheimer's disease, neuron

## Abstract

Multicellular signals are altered in the processes of both aging and neurodegenerative diseases, including Alzheimer's disease (AD). Similarities in behavioral and cellular functional changes suggest a common regulator between aging and AD that remains undetermined. Our genetics and behavioral approaches revealed the regulatory role of Akt in both aging and AD pathogenesis. In this study, we found that the activity of Akt is upregulated during aging through epidermal growth factor receptor activation by using the fruit fly as an in vivo model. Downregulation of Akt in neurons improved cell survival, locomotor activity, and starvation challenge in both aged and Aβ42‐expressing flies. Interestingly, increased cAMP levels attenuated both Akt activation‐induced early death and Aβ42‐induced learning deficit in flies. At the molecular level, overexpression of Akt promoted Notch cleavage, suggesting that Akt is an endogenous activity regulator of γ‐secretase. Taken together, this study revealed that Akt is involved in the aging process and Aβ toxicity, and manipulating Akt can restore both neuronal functions and improve behavioral activity during the processes of aging and AD pathogenesis.

## 1 INTRODUCTION

Aging‐associated physiological changes are common to all species, from yeast to mammals, and considered an irreversible process involving changes in multicellular signals. Because neurons lack the ability to self‐regenerate and are sensitive to stressors (e.g., oxidative stress or nutrition deficit), they are extremely vulnerable to aging damage (Mattson & Magnus, 2006). Degenerated neurons in the brain have been associated with many physiological and pathological changes. Accumulated evidence suggests that age is the strongest risk factor for neurodegenerative diseases, including Alzheimer's disease (AD). Molecular signals found to be changed during aging are sometimes also altered in neurodegenerative diseases (Colacurcio & Nixon, 2016; Jagust, 2013; Winick‐Ng & Rylett, 2018). There are many similarities between an aged animal and an animal with a neurodegenerative disease; for instance, (a) accumulated oxidative stress is observed in both animals (Hernández‐Camacho, Bernier, López‐Lluch & Navas, 2018; Hussain et al., 2018; Prolla & Mattson, 2001), (b) the requirement of nutrition is increased (Harding, Gonder, Robinson, Crean & Singhrao, 2017; Prolla & Mattson, 2001), (c) learning and memory performance decline, and (d) multicellular signals are altered in the brain. However, whether there is one common cellular pathway that links and regulates both aging‐related pathologies and neurodegenerative diseases remains unclear.

The Akt (protein kinase B) signaling pathway is involved in both aging and AD. Aged brains were found to be associated with an imbalance of phosphatidylinositide 3‐kinases (PI3k)/Akt signaling (Jackson, Rani, Kumar, & Foster, 2009; Jiang, Yin, Yao, Brinton & Cadenas, 2013; Yang et al., 2018). In addition to the alteration of PI3K/Akt signaling in the aged brain, increased Akt phosphorylation is also observed in other aging tissues. For example, hyperactivation of Akt is associated with aging in muscle cells (Wu et al., 2009). In addition, aging was shown to increase Akt phosphorylation in murine heart tissues (Hua et al., 2011). Expected animal lifespan can be affected by the Akt pathway. Interestingly, an increased expression of the *Drosophila* forkhead transcription factor (dFOXO) in the fat body, but not neurons, inside a brain extended lifespan (Hwangbo, Gershman, Tu, Palmer & Tatar, 2004). The suppression of insulin‐induced Akt signaling in *Caenorhabditis elegans* increased their lifespan (Dillin, Crawford & Kenyon, 2002). Overactivated Akt was also found in the brain of AD (2003, Rickle et al., 2004; Griffin et al., 2005). The genomewide analysis of miRNA in AD mouse models showed altered Akt expression (Luo et al., 2014). Interestingly, decreased PI3k activity has been shown to be capable of reversing Aβ42‐mediated learning impairment (Chiang, Wang, Xie, Yau & Zhong, 2010). Although accumulated evidence suggests that PI3k/Akt signaling participates in both aging and AD, the detailed underlying mechanism is still not completely understood. Whether the PI3k/Akt signaling pathway governs both aging‐related pathologies and AD pathogenesis and whether aging‐ and AD‐induced damages can be alleviated by targeting this pathway require further investigation.

This study employed *Drosophila melanogaster* as an animal model that has been used to investigate the aging process and AD for decades (Iijima et al., 2004; Prüßing, Voigt & Schulz, 2013). Results of our study revealed that Akt activity is increased in aged animals. Importantly, we demonstrated that overexpression of dFOXO reversed Aβ42‐induced learning deficit. We further demonstrated that increased cAMP levels reversed Akt‐induced behavior deficits, both in aged and in Aβ42‐expressing animals. In this study, we also found that Akt regulates γ‐secretase activity and APP processing, suggesting that Akt mediates the link between aging and AD. This study reveals a critical role of Akt in the aging process, AD pathogenesis, and Aβ toxicity and provides mechanistic insights into the development of future therapeutic strategies to reverse or delay aging‐related pathology.

## 2 RESULTS

### 2.1 Decreased Akt expression in neurons reverses most aging‐related pathologies

To prevent developmental defects caused by genetic manipulation, unless mentioned otherwise, the conditional expression system Gal80^ts^ was used in this study (McGuire, Mao & Davis, 2004). Flies were moved from 18 to 30°C after eclosed to fully express target genes. All *UAS‐target* genes were driven by *Elav‐Gal4*, which is a known pan‐neuronal driver.

**Figure 1:**
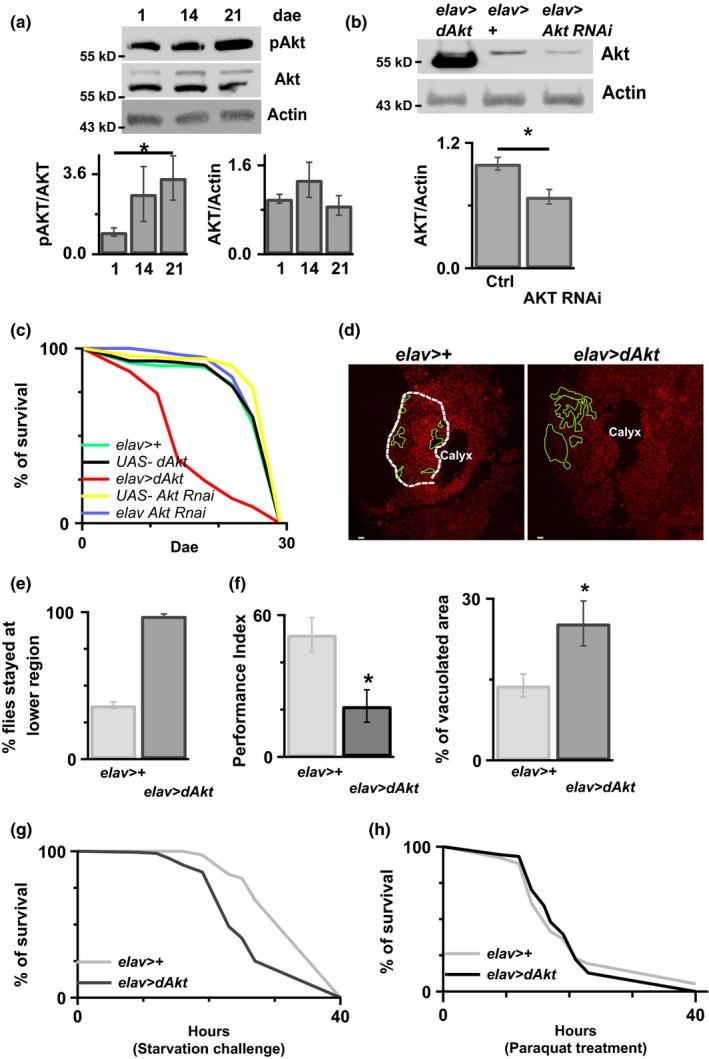
Increased Akt activation during aging reduces animal survival. (a) Flies were collected at three different ages (1, 14, and 21 dae), and expression of different proteins was determined by the Western blot analysis. Actin was used as an internal control. The intensity of each band was quantified, and graphs showing the relative band intensity are included. *=*p *<* *0.05. *n* = 8–11. (b) After 7 days in the 30°C to induce AKT or AKT RNAi, there was increased or decreased AKT expression level, respectively. *N* = 11. *=*p* < 0.01. (c) The survival period of control flies, flies without genetic manipulation of Akt, and flies with either overexpression of Akt or downregulation of Akt in neurons was measured using lifespan assay. (d) Cell death in the region of Kenyon cell body of 7 dae flies with or without Akt overexpression was determined using fluorescence microscopy. The area measured was circled in white. Areas with no clear PI staining (indicates cell lost) were circled in green white bar = 2 μm. Percentage of vacuolated area within calyx was quantified and shown in the right panel. *=*p *<* *0.05. *n* = 10–11. (e) The climbing ability of flies with or without Akt overexpression in neurons was evaluated in the 10‐day‐old flies. (f) Learning deficit was observed in the flies with Akt overexpression compared to the control group. The performance index was lower in the *elav*>*Akt* flies than *elav*>*+* flies. *=*p *<* *0.05. *N* = 5–7. (g) Starvation challenge, flies with Akt overexpression showed less resistant to the starvation challenge compared to the flies without Akt overexpression, *N* = 75 and 140, for *elav*>*+* and *elav*>*Akt*, respectively. Time zero indicates the beginning of starvation. (h) Paraquat challenge, there was no effect on the response to the oxidative stress in the Akt flies compared to the control. The survival rate was similar between *elav*>*Akt* flies and *elav*>*+* flies after paraquat treatment. *N* = 155–165

**Figure 2:**
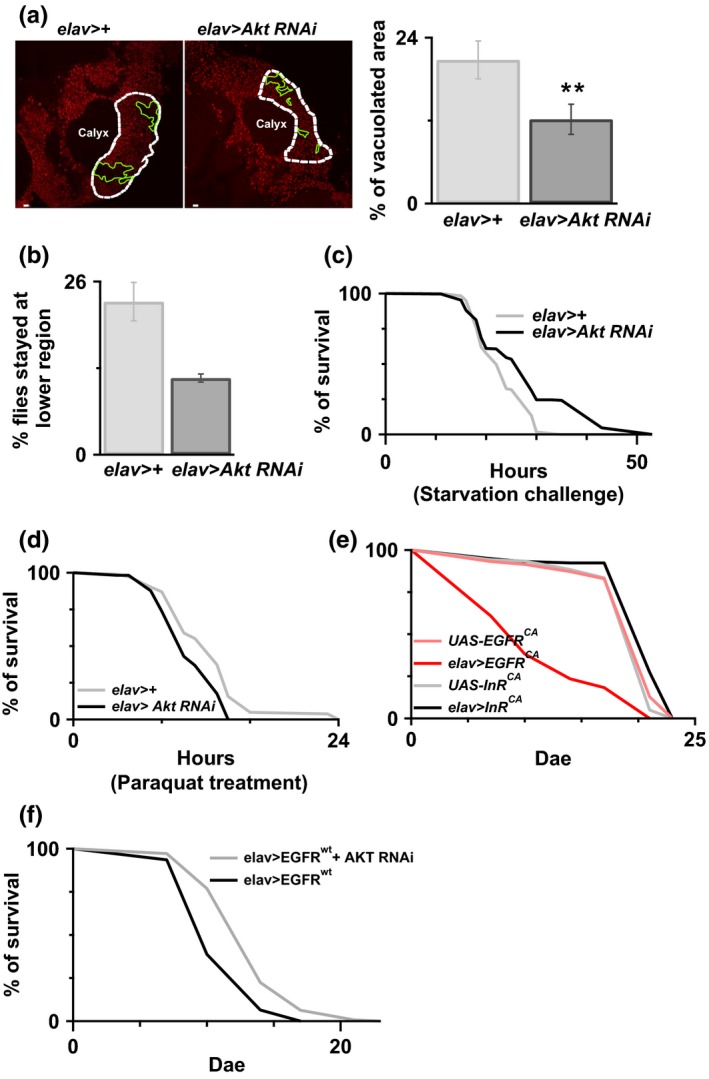
Reduced Akt benefits animal survival. (a) Reduced Akt in neurons by RNAi promotes cell survival in the Kenyon cell body region at the age of 20 days. White dot circle is the place where we used to do the quantification and is above the calyx. Green circles represent the area where there was no clear PI staining, which suggests there were cells lost. Percentage of vacuolated area (i.e. percentage of area circled in green) within calyx was quantified and shown in the right panel. *N* = 5–7. **p <* *0.01. White bar = 2 μm. (b) A better climbing ability was observed in the Akt knocked‐down flies compared to the control flies, without RNAi expression. Less Akt RNAi flies stay at the bottom. (c) Reduced Akt in neurons showed better starvation resistance. The survival rate of *elav*>*Akt RNAi* was better than *elav*>*+*. *p* < 0.001 log‐rank analysis. *N* = 180 for *elav*>*+* and *N* = 270 for *elav*>*Akt RNAi*. (d) Reduced Akt has no any improvement on the oxidative stress. The survival rate between flies, with/without Akt knocked‐down, was similar. *N* = 180 for *elav*>*+* and *N* = 110 for *elav*>*Akt RNAi*. (e) Overexpression of EGFR^CA^ but not InR^CA^ in the flies promoted animal early death compared to the control flies, flies without any genetic manipulation. *N* = 120 for each genotype. There was significant lifespan decreasing in the flies with EGFR^CA^ overexpression. *p* < 0.001 log‐rank analysis. (f) Reduced AKT delays EGFR ^wt^‐induced early death. *N* = 140–200. *p* < 0.001 log‐rank analysis

Fruit flies have widely been used to assay longevity and aging‐related damages, such as stress resistance, locomotion ability, and cell survival (He & Jasper, 2014; Jones & Grotewiel, 2011). To determine the potential roles of Akt in aging, we first examined the level of Akt activation, phosphorylation Akt (pAkt), in both aged and control flies. As shown in Figure 1a, the amount of phosphorylated Akt was significantly increased in aged flies, the 21 days after eclosion (dae) flies, as compared to control flies, the 1‐dae flies, indicating that Akt activation may play a role in aging. To understand the role of Akt in aging, we genetically manipulated the Akt expression level in transgenic flies, Figure 1b and Fig. S1. As shown in Figure 1c, overexpression of Akt promoted early death in flies. In contrast, downregulation of Akt by RNAi showed no effect on longevity. Propidium iodide staining (PI stain) has been used for examining cell death in other work (Iijima et al., 2004). Results of the fluorescence microscopy showed that Akt overexpression promoted early cell death, as indicated by less PI stain positive signals in the Kenyon cell body region (Figure 1d). Behavioral studies revealed that Akt overexpression also caused climbing impairment (Figure 1e), learning deficit (Figure 1f), and reduced resistance to the starvation challenge (Figure 1g) in flies. However, no effect on oxidative stress resistance was observed in the Akt overexpressing flies (Figure 1h). In contrast, downregulation of Akt by RNAi reduced the amount of cell death in the Kenyon cell body region (Figure 2a), improved climbing activity (Figure 2b), and increased resistance to starvation (Figure 2c). Similar to the results of flies with Akt overexpression, Akt downregulation did not affect the resistance to oxidative stress in the aged flies (Figure 2d).

Akt is a downstream signaling molecule of both the epidermal growth factor receptor (EGFR) and insulin receptor (InR) signaling pathways. Here, we overexpressed the constitutive active mutant, EGFR^CA^ (A887T) or InR^CA^ (R418P) in the neurons. Interestingly, only flies with neurons with EGFR^CA^ overexpression showed early death (Figure 2e). Similar to the results of flies with neuronal EGFR^CA^ and InR^CA^ overexpression, only flies with EGFR^WT^, but not InR^WT^, overexpression showed early death (Fig. S2). Moreover, we observed that AKT downregulation delayed EGFR‐induced early death, Figure 2f. We also confirmed that overexpression of InR in the neurons is able to increase Akt phosphorylation in the older flies, 14 dae, which suggests the specificity of EGFR downstream signaling (Fig. S3). These results suggested that the EGFR‐Akt signaling in neurons plays a role in the causation/promotion of aging‐like pathology, at least for the early death.

### 2.2 Overexpression of dFOXO, a downstream molecule Akt, attenuates Aβ‐induced early death in flies

**Figure 3:**
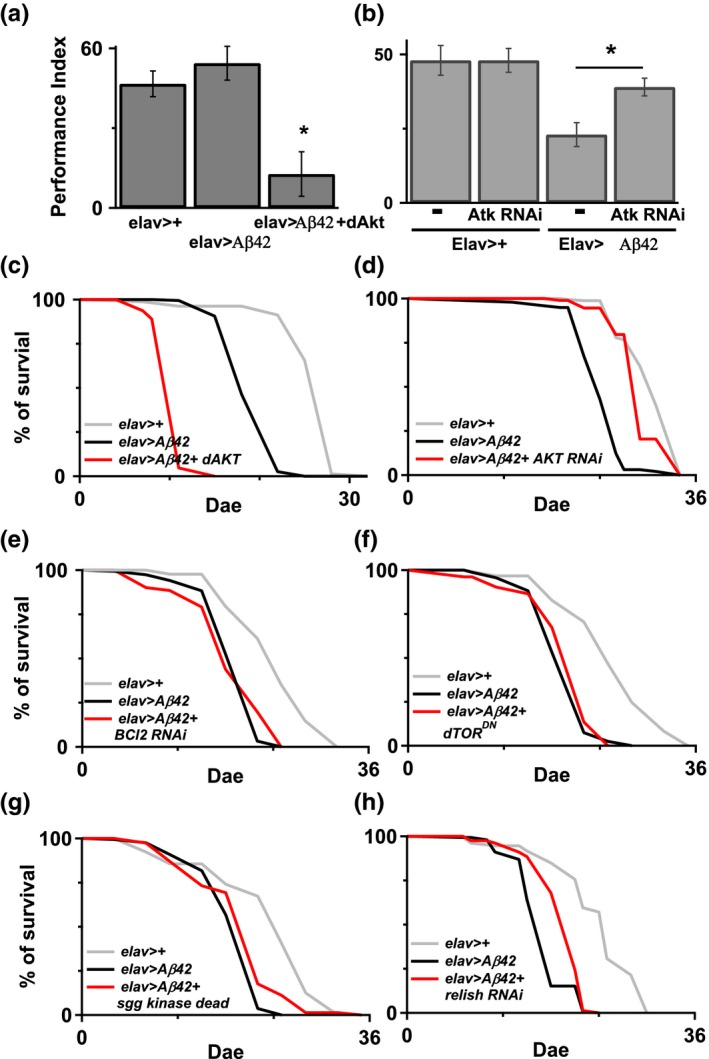
Genetic manipulation of Akt is able to regulate Aβ42‐induce longevity impairment. (a and b) Coexpression of Akt and Aβ42 showed a significant damage in the transgenic animals. *p < 0.05. *N* = 6–7. Neuronal Akt downregulation in the Aβ42 flies reversed Aβ42‐induced learning deficit. *N* = 6. (c and d) Overexpressed Akt in the Aβ42 flies, *elav*>*Aβ42 + dAKT*, enhances animal early death, while flies with reduction of Akt, *elav*>*Aβ42 + dAKT RNAi*, reversed Aβ42‐induced lifespan shortening compared to the Aβ42 flies. For c, *N* = 160–190. For d, *N* = 90–160. There was significance between *elav*>*Aβ42* and *elav*>*Aβ42 + Akt RNAi*. *p* < 0.001 log‐rank analysis. (e and f) Genetic expression of Bcl‐2 RNAi, and dTOR^DN^ in neurons in the Aβ42 flies had no improvement on Aβ42‐induced early death in the *elav*>*Aβ42* flies, whereas overexpressed Shaggy mutant or reduced relish by RNAi improved Aβ42‐induced early death in the *elav*>*Aβ42* flies, g and h. *N* = 80–180

**Figure 4:**
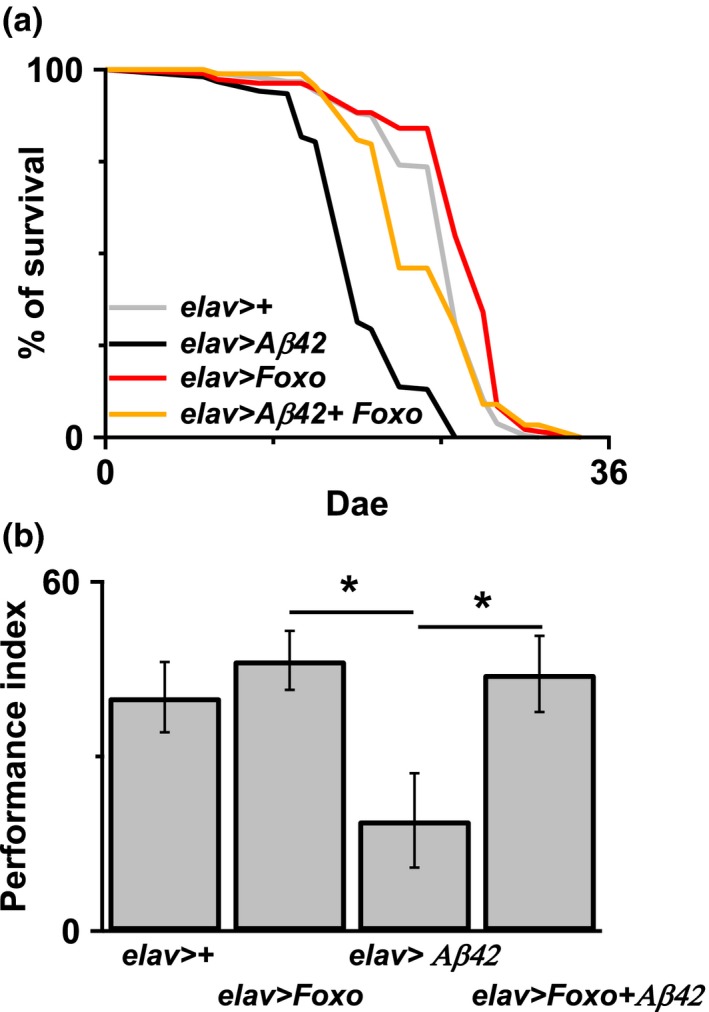
Overexpressed FOXO improved Aβ42‐induced learning deficit and early death. (a) Overexpressed FOXO in neurons in the Aβ42 flies significantly reversed Aβ42‐induced early death in the flies. *p* < 0.001 between *elav*>*Aβ42* and *elav*>*Aβ42 + FOXO*. *N* = 90–180. (b) Learning deficit induced by Aβ42 overexpression in the 7‐ to 8‐day‐old flies can be reversed by FOXO overexpression in the same neurons. *p < 0.05. *N* = 9–13

AD is the most common form of dementia and is the leading neurodegenerative diseases. Genetic studies have shown a causative link between Aβ peptides and AD. Previous studies have demonstrated that decreased EGFR–PI3K signaling could reverse Aβ‐induced learning impairment in Aβ42 flies (Chiang et al., 2010; Wang et al., 2012). Therefore, we speculated that upregulation of Akt might also mediate Aβ‐induced pathology. In our study, overexpression of Akt in Aβ42 flies enhanced learning deficit (Figure 3a), and there is no observed learning deficit in 5‐day‐old Akt flies (Fig. S4A) and caused their early death (Figure 3c), whereas downregulation of Akt improved learning performance (Figure 3b) and increased the survival period in Aβ42 flies (Figure 3d). Of note, inhibiting Akt activity with pharmacological inhibitor MK2206 (100 μM) also improved Aβ42‐induced early death and learning deficit (Fig. S4B, C). We subsequently investigated the downstream signaling pathway of Akt, which can be responsible for the Aβ42‐induced toxicity, by manipulating genes that were downstream of the Akt signaling pathway in Aβ42 flies. Downregulation of buffy, a *Drosophila* Bcl‐2 protein (Figure 3e), and overexpression dominate negative Tor, and TOR^DN^ (Figure 3f) had no effect on Aβ42‐induced early death in flies. Although there was an improvement in survival with the overexpression of Shaggy mutant, a *D*. *melanogaster* orthologue of GSK3β (Figure 3g), and the downregulation of Relish, a *Drosophila* NF‐κB/IκB protein (Figure 3h), overexpression of dFOXO exerted the greatest effect on reducing Aβ42‐induced early death in flies (Figure 4a). Overexpressed dFOXO in flies also improved Aβ42‐induced learning impairment (Figure 4b).

### 2.3 Akt is an endogenous γ‐secretase activity regulator

**Figure 5:**
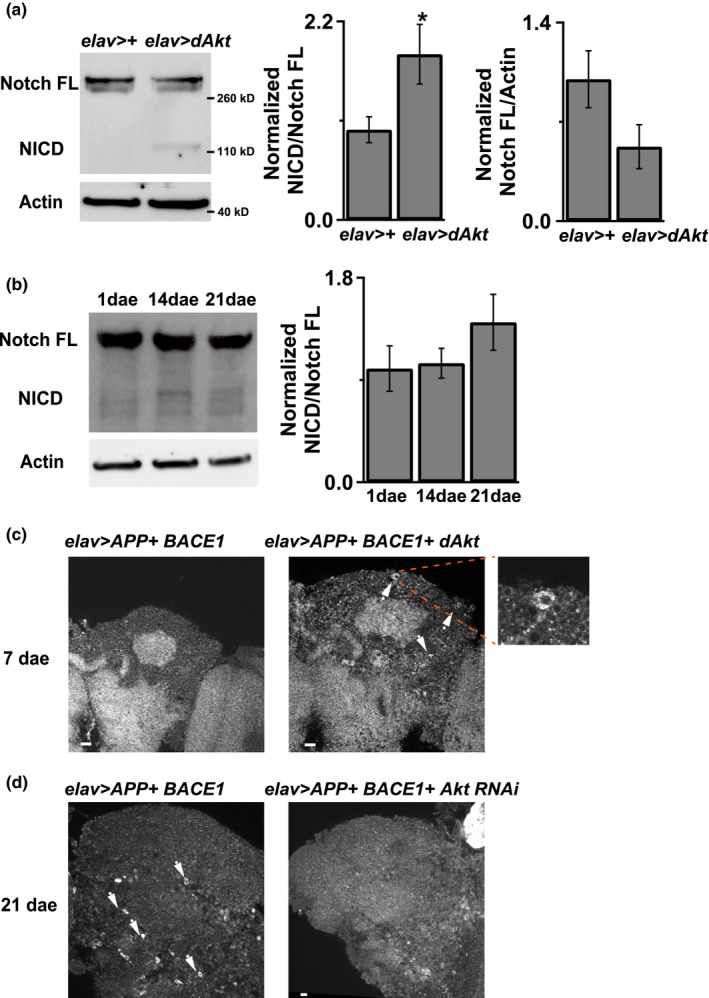
Akt regulates Notch and APP processing. (a) Akt overexpression in neurons, *elav*>*Akt,* promoted endogenous Notch processing. 7‐ to 8‐day‐old flies’ brain was used to perform Western blot analysis. There was more NICD band intensity in the Akt overexpressed flies. *N* = 8 for each genotype. *p < 0.05. (b) Western analysis showed that 21‐day‐old wild‐type fly had more endogenous NICD in the brain than 1‐day‐old fly. *p* < 0.12. *N* = 8–9. (c) Overexpressed Akt in neurons in the APP+ BACE1 transgenic flies showed more positive immunofluorescent signals, 6e10 antibody staining, surrounding cell body than control flies, APP+ BACE1 flies but no genetic manipulation of Akt, which indicated more APP processing. Arrowhead indicated positive signals. (d) Reduced Akt in neurons decreased the number of immunofluorescent signals, 6e10 antibody staining, in the APP+ BACE1 transgenic flies. Arrowhead indicated positive signals with cell shapes. Mushroom‐body area was used for images analysis. White bar = 10 μM. *N* = 3–4

Aging is the strongest risk factor for AD, and our results revealed that the activity of Akt is upregulated in aged flies. As the Aβ peptide is a cleavage product of amyloid precursor protein (APP); therefore, we hypothesized that Akt is involved in AD pathogenesis, possibly through modulations of the activity of secretase. Since Notch is an endogenous γ‐secretase substrate in flies, we examined the endogenous γ‐secretase activity by measuring the level of Notch cleavage in flies with or without Akt overexpression. The intracellular domain of the notch protein (NICD) is a Notch cleavage product generated by γ‐secretase. Here, results of the Western blot analysis revealed the presence of NICD in the Akt overexpressed flies but not in the control flies (Figure 5a). This result suggested that Akt is the endogenous γ‐secretase regulator to regulate Notch activity. Intriguingly, we also found a trend of increasing NICD productions in aged flies, despite the result did not reach statistical significance (*p *<* *0.12; Figure 5b).

We then investigated whether Akt affects APP processing. We double overexpressed the full‐length APP and BACE1 in flies (Chakraborty et al., 2011) with different Akt expression manipulations (either downregulation or overexpression). Since we observed an increased in immune‐fluorescence signal (Aβ antibody staining) surrounding the neuronal cell bodies in the Akt transgenic flies as compared to the control flies (Figure 5c), we focused on the mushroom‐body region as this area is the learning and memory center for a fruit fly. Here, immunostaining assay was performed on tissue sections of 7 dae flies, because overexpression of Akt causes early death and we wanted to avoid inducing severe death of flies prior to the experiment. Results of the immunofluorescence microscopy revealed a decrease in fluorescence signal surrounding neuronal cell body in the 21 dae Akt‐downregulated flies (Figure 5d). Taken together, these results indicate that Akt is a γ‐secretase regulator capable of affecting the APP processing.

### 2.4 Increased cAMP reduces the risk of Akt‐induced early death

**Figure 6:**
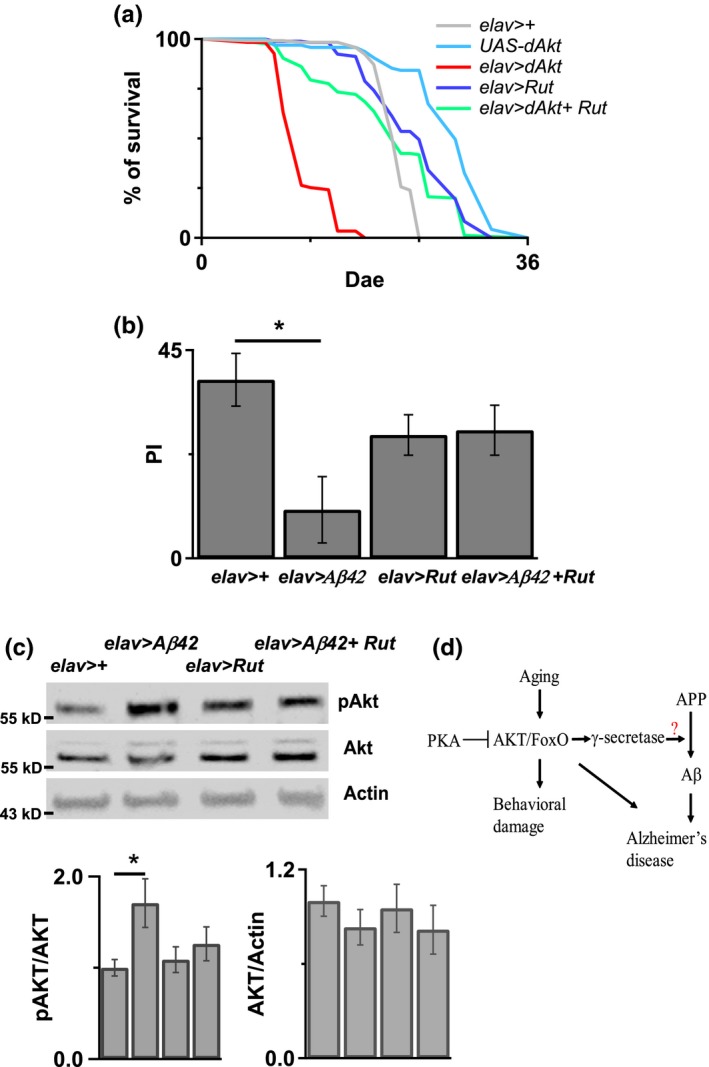
Overexpressed Rutabaga reversed Akt‐induced early death and Aβ42‐induced Akt activation and learning deficit. (a) Overexpressed Rutabaga in neurons reduced Akt‐induced early death in the *elav*>*Akt* flies. *N* = 100–170. There was significance between *elav*>*Akt* and *elav*>*Akt+ Rut*. *p* < 0.0001. Log‐rank analysis. (b) Rutabaga overexpression in neurons reversed Aβ42‐induced learning deficit in the 7‐ to 8*‐*day‐old *elav*>*Aβ42* flies. *N* = 6 for each genotype. *p < 0.05. (c) Western blot showed Rutabaga overexpression in neurons reduced Aβ42‐induced Akt phosphorylation in the 7‐ to 8‐day‐old *elav*>*Aβ42* flies. Bottom, the quantitative results showed that the Western blot band intensity of pAkt in *elav*>*Aβ42 + Rut* flies was less than in *elav*>*Aβ42* flies. *N* = 9–14. *p < 0.05. This experiment was done at 25°C without heat induction. (d) Aging promotes Akt activation, which induces behavior damage and activates APP processing to promote Alzheimer's disease formation

Previous study showed that cAMP treatment prolonged the lifespan of fruit flies (Tong, Schriner, McCleary, Day & Wallace, 2007). In addition, upregulation of intracellular cAMP by inhibiting the cAMP‐degrading phosphodiesterases was shown to be able of attenuating aging‐related metabolic diseases (Park et al., 2012). To identify signaling pathways that could be used to reduce the risk of Akt‐induced early death, we evaluated the effect of cAMP on Akt‐induced early death. As shown in Figure 6a, pan‐neuronal expression of the wild‐type rutabaga (Ca^2+^/calmodulin‐responsive adenylyl cyclase) reduced the early death rate in Akt transgenic flies. Furthermore, rutabaga overexpression also reversed the learning deficit in Aβ42 flies (Figure 6b). To strengthen our finding that Akt signaling is the potential link between age and AD, we have shown that the learning deficit was found in flies with overexpression of both Aβ42 and Akt in 5‐day‐old flies (Figure 3a). To confirm our hypothesis that increased cAMP can reduce Akt activity in Aβ42 flies, we examined pAkt levels in Aβ42 flies. We found that overexpression of rutabaga decreased the expression of pAkt in Aβ42 flies (Figure 6c). Collectively, these results suggest that Akt is involved in both aging and the development of AD, and the reversal of Akt overexpression by cAMP can alleviate both aging‐induced and Aβ42‐induced pathologies.

## 3 DISCUSSION

The current study revealed that aging upregulates Akt activity to trigger aging‐related pathologies, such as decreased longevity, less resistance to stress, locomotion deficit, cell death, and learning and memory deficit. In contrast, Akt downregulation in the brain not only mitigates these pathological phenotypes but also the Aβ‐induced damage. Therefore, our data suggest that Akt participates in both the aging process and Aβ toxicity. Moreover, our findings revealed that Akt is an endogenous activity regulator of γ‐secretase in the brain. We found that increased cAMP could overcome Akt activation‐induced damages, including Aβ‐induced learning deficit. Therefore, we propose two methods to inhibit aging‐like impairments: reduction of Akt and elevation of cAMP (Figure 6d).

Akt has long been though as a survival factor in a wide range of cell types. Thus, findings of this study seem to contradict to the procell survival role of Akt. However, most functional investigations on Akt were in vitro studies carried out under pathological conditions; functional role of Akt has relatively less been studied in vivo under physiological conditions. Of note, reduced insulin signal transduction, an upstream of Akt, has been shown to prolong animal longevity in different animal models (Bluher, Kahn & Kahn, 2003; Dillin et al., 2002; Huang et al., 2015). Therefore, it is possible that Akt exhibits differential molecular and cellular functions in different cell types and during different physiological and pathological development stages. Hwangbo et al. (2004) found that dFOXO overexpression in the brain surrounding fat body, but not in neurons, of fruit flies extended their lifespan and promoted oxidative stress resistance. Results of our study not only in line with their findings but also suggest that Akt plays differential roles in different cell types within the brain. In fact, downregulation of Akt in neurons only improved the locomotion and performance in the starvation tests but not the longevity and resistance in the oxidative stress. Furthermore, increased EGFR, but not InR, mediates these effects in the neurons, at least in affecting animal longevity. In fact, other works have also reported that downstream signaling that EGFR activates Akt is not exactly the same as InR actives Akt (Borisov et al., 2009; Kumar, Afeyan, Sheppard, Harms & Lauffenburger, 2007; Roudabush, Pierce, Maudsley, Khan & Luttrell, 2000). Although the molecular reason behind the differential functions of Akt in different cell types is unclear, the cell type‐specific molecular property of Akt may provide a means of differentially and precisely regulating the functions of various areas of the brain under different physiological conditions.

Accumulated evidence supports that cAMP and cAMP‐related signaling pathway are involved in the aging process. For example, fruit flies with increased cAMP showed extended lifespans (Tong et al., 2007). Moreover, the average lifespan of mice treated with cAMP was prolonged by 6 weeks as compared to that of the untreated mice (Wang et al., 2015). In our study, we did not find any significant increase in the lifespan of flies with rutabaga overexpression. However, we found a reduced early death rate in flies with Akt overexpression. Given that the activity of Akt is upregulated during aging, results of our study are in line with previous findings that increased cAMP can attenuate the cellular and behavioral effects of the aging process. It is also worth noting that we also observed an inverse correlation between the overexpression of rutabaga and the activity of Akt in the Aβ42 flies, further supporting our findings. It has been demonstrated that cAMP/PKA inhibits Akt activity (Kim, Jee, Kim, Koh & Chung, 2001; Lou, Urbani, Ribeiro‐Neto & Altschuler, 2002). As Aβ42‐induced Akt activation plays an important role in mediating Aβ42 toxicity and increased cAMP can improve the learning performance through reducing Akt activation in the Aβ42 flies, strategies like reducing Akt signaling and increasing cAMP levels can probably be used to reverse or to delay the progress of the aging‐related pathologies. Although we do not know how cAMP affects Akt‐induced impairment, we believe that this effect is unlikely caused by direct interaction between these two molecules. Noticeably, we did not find any significant decrease in Akt activity after rutabaga overexpression. Therefore, cAMP signaling pathway may regulate the downstream signaling of Akt.

Multicellular signals, including PI3K/Akt, are altered in brains with AD (Griffin et al., 2005; Rickle et al., 2004). AD animal with reduced PI3K/Akt has been shown to exhibit better behavior performance in different species (Chiang et al., 2010; Wang et al., 2012). Akt and its downstream signals are essential for the maintenance of normal cellular functions. Although genetically manipulating various Akt downstream signaling molecules has been shown to exert positive effects on reversing Aβ toxicity, most studies were conducted on different animal models, making it difficult to determine targeting which of the Akt downstream signaling molecules exerts the highest protective effect against Aβ‐induced toxicity (Jones & Kounatidis, 2017; Llorens‐Martín, Jurado, Hernández & Avila, 2014; Norambuena et al., 2017). Our genetic manipulation studies showed that targeting NF‐κB and dFOXO‐related pathways produce positive protective effects on Aβ‐induced pathology. Nf‐κB downregulation or dFOXO overexpression both extended the lifespans of Aβ42 flies. However, the protective effects of dFOXO overexpression were more prominent than those of Nf‐κB downregulation in Aβ42 flies. Consistent with this result, dFOXO overexpression reversed Aβ42‐induced learning deficit, supporting that manipulation of the FOXO signaling pathway can reduce Aβ42 toxicity and also warranting further investigations for the development of its modulators for the treatment of AD.

Increased Aβ accumulation is observed in the aged brain and is considered as one of the main causes of late‐onset AD. Until now, only a few conditions (like oxidative stress and lipid peroxidation) and molecules (like SGK1 [serum‐ and glucocorticoid‐inducible kinase 1], and ERK 1/2) have been identified to be capable of affecting/regulating the γ‐secretase (Guix et al., 2012; Gwon et al., 2012; Kim et al., 2006; Mo et al., 2011). In our study, we found that Akt regulates Notch cleavage, suggesting that Akt is one of the endogenous γ‐secretase regulators. Because aging promotes Akt activation, our results further support that age contributes largely for the development of AD and provide a molecular mechanistic explanation for the importance of aging in the development of late‐onset AD. A positive feedback loop between Akt and the AD process may also be existed. Aging increases Akt activity to promote γ‐secretase activity, which could lead to APP cleavage and generate Aβ, and the increased Aβ activates more Akt to further increase γ‐secretase activity and to promote APP processing. At this point, we are unsure whether Akt regulates γ‐secretase activity directly or indirectly and also which γ‐secretase subunit is regulated by Akt. Whether Akt activity upregulation during aging can be used as an early biomarker to indicate the probability of Aβ production and the incidence of sporadic AD or as a preventative treatment against AD requires further investigations.

As senior populations are increasing globally, aging‐related pathologies are receiving more attention. Because of the increased economic burden on health care, it is of clinical importance to understand aging and its related pathologies. The dual roles of Akt in aging‐related pathologies and AD suggest that Akt signaling is an attractive molecular target for research and treatment. We have showed that tissue‐specific signaling can activate Akt, demonstrating how the brain regulates cellular function in different areas to meet physiological needs with the same downstream signal and provides a more flexible strategy to reverse aging‐related pathologies. Aging is the leading risk factor for AD, but it remains unclear on how aging is involved in the pathogenesis of late‐onset AD. Our study revealed a correlation between Akt activation and γ‐secretase activity. This finding provides a molecular explanation on how aging increases the incidence of AD, especially for the development of late‐onset AD. The development of strategies to prevent Akt activation warrants further investigation.

## 4 MATERIALS AND METHODS

### 4.1 Fly strains

*UAS‐FOXO* (9575)*, UAS‐EGFR* ^*CA*^ *(9533), UAS‐EGFR* ^*wt*^ *(5368), UAS‐InR* ^*CA*^ *(8250), UAS‐InR* ^*wt*^ *(8262), UAS‐dTOR* ^*DN*^ (7013), *UAS‐sgg* ^*kD*^ (8712), *UAS‐rutabaga, UAS‐APP, and UAS‐BACE1* (33798) were from Bloomington stock center. *UAS‐BCl2*

*RNAi, UAS‐NF*κ*B RNAi* were from VDRC. *UAS‐Aβ42* (Iijima et al., 2008), *Elav‐Gal4* ^*c155*^, *UAS‐dAKT, tubulin‐Gal80* ^*ts*^ are kindly provided by Dr. Yi Zhong at Tsing‐Hua University, China. *UAS‐Akt RNAi* was from Tsing Hua Fly center (Ni et al., 2008, 2011).

### 4.2 Pavlovian olfactory aversive conditioning

Training and test were performed as described previously (Tully & Quinn, 1985). In brief, flies were placed in an electric shock tube during training and were exposed to two odors (3‐octanol [OCT] and 4‐methylcyclohexanol [MCH] which concentrations adjusted by daily condition to avoid bias) and electric shocks. Animals were exposed to OCT or MCH (conditioned stimulus, CS+) pairing with foot electrical shock (unconditioned stimulus, US) (90 mV) for 1 min, then receive the other odor (CS−) without foot shock for 1 minute. There were 45 seconds of rest between CS+ and CS−.

During the test for immediate short‐term memory (also referred to as learning), trained flies were immediately transferred into a T‐maze where they allowed to choose between CS+ and CS− for 2 min. The distribution of flies in the two arms of T‐maze was referred to as performance index, flies in the CS− odor minus flies in the CS+ odor and divided by total flies. A PI of 0 means there is no learning, and distribution of flies in the CS+ and CS− is 50:50. A PI of 100 means all the flies stayed in the CS+. To prevent odor bias, a performance index was collected from two reciprocal groups. One trained to associate one odor, the other group was trained to associate the other odor. Control groups are age‐matched to the experimental groups in each test.

### 4.3 Survival assay

The lifespan studies were performed more than three times. We changed the food and counted flies every 3–4 days. The experiments were placed in 18 or 30°C (adjusted by experimental condition), 70% humidity, with a 12‐hr light/dark cycle.

### 4.4 Western blot analysis

Collected 5 fly heads were homogenized in SDS sample buffer, run on 10–20% Tris–Tricine gels, and transferred to nitrocellulose membranes (PALL). Anti‐AKT and pAkt antibodies were purchased from cell signaling. Notch antibody was from hybridoma. Data were analyzed with software ImageJ (National Institutes of Health).

### 4.5 Immunohistochemistry and Immunofluorescence

Flies were quickly dissected in the phosphate‐buffered saline (PBS). Brains were fixed and permeabilized in 4% (wt/vol) paraformaldehyde in PBS. They were mounted within FocusClear, and coverslips were added. 6E10 antibody (BioLegend) against Aβ was used to detect Aβ deposition. Slides were inspected with a confocal laser scanning microscope (FV1000).

For PI staining, before confocal exposure, brains were incubated with 1:200 propidium iodide at 4°C overnight. The ratio of the area of vacuoles in the mushroom‐body cell body region was calculated by dividing the sum of the vacuole areas by the total area of the cell body region. All statistics were calculated from three or more brains. As indicated in the figure, we measured the area above the calyx, mostly Kenyon cell body area. In order to prevent any bias, all the analysis was done by blind analysis. People who did the analysis did not know the genotypes.

### 4.6 Locomotion assay

Empty vial with 9 cm high and 3 cm wide was used to do the assay. Each vial was labeled three parts, top, middle, and bottom. Each empty vial contains around 25 flies. 10 seconds after gently tapped, the number of flies that stayed at the bottom part will be recorded. Each experiment was repeated three times.

### 4.7 Stress resistance assay

Stress resistance (oxidative stress and starvation) was determined at 5‐day‐old flies with Akt overexpression and 16‐ to 20‐day‐old Akt knocked‐down flies paired with control group. Flies were kept in the vial with 3M filter paper soaked with 4% sucrose solution contained 40 mM paraquat solution for oxidative stress assay. Starved flies were kept in the empty vial with moistened paper.

### 4.8 Statistics

All data were analyzed using GraphPad Prism 6.0 software. Comparisons between two groups used two‐tailed *t* test. Comparisons of multiple groups used one‐way ANOVA. Lifespan analysis was done by using log‐rank test. Statistical significance was shown with *p* value < 0.05. Statistical results are presented as means ± *SEM*.

## CONFLICT OF INTEREST

None declared.
