# Supplementary material for: Aging‐induced Akt activation involves in aging‐related pathologies and Aβ‐induced toxicity
Source: Aging Cell. 2019 Jun 11;18(4):e12989. doi: 10.1111/acel.12989 (PMC6612704; doi:10.1111/acel.12989)
Supplement: Supplementary file 1 [file ACEL-18-e12989-s001.docx]

Supplementory Figures


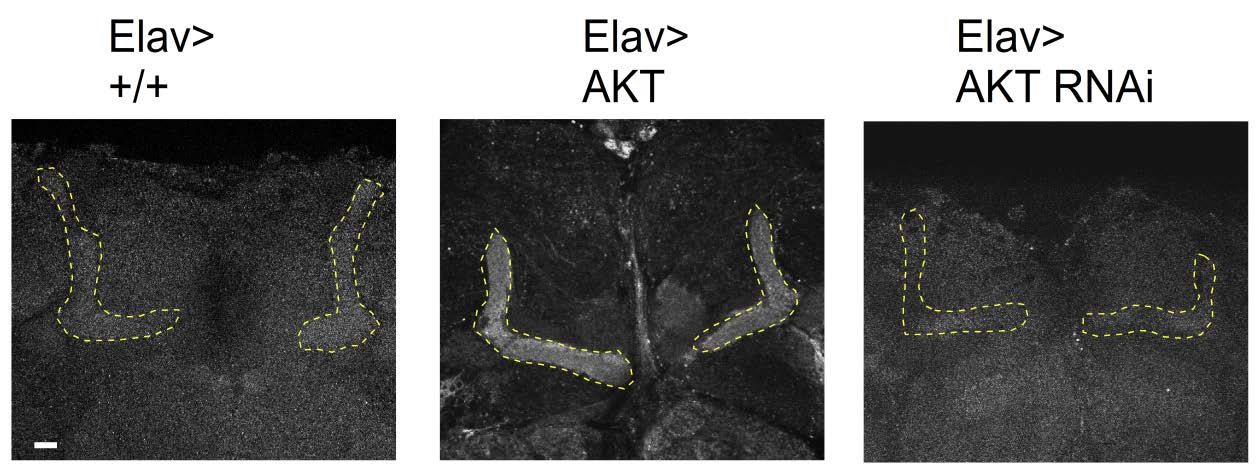


Fig. S1. Overexpressed AKT or AKT RNAi changes AKT expression level. Representative results of immunostaining. Anti-Akt antibody was used for immunostaining. Yellow dots means mushroom body lobes. N=3-5. White bar= 4_µ_m.


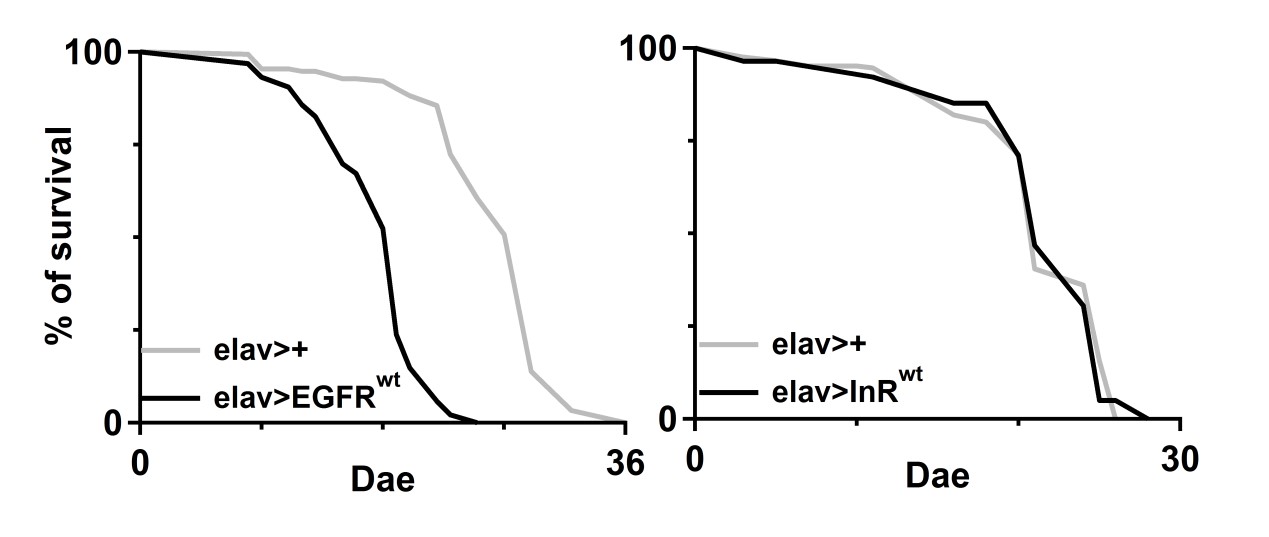


Fig. S2. Neuronal expression of wild-type EGFR induced early death. Transgenic flies overexpressed with EGFR triggered animal early death. There is significance between elav>+ and elav>EGFR^wt^. P<0.001 log-rank analysis.


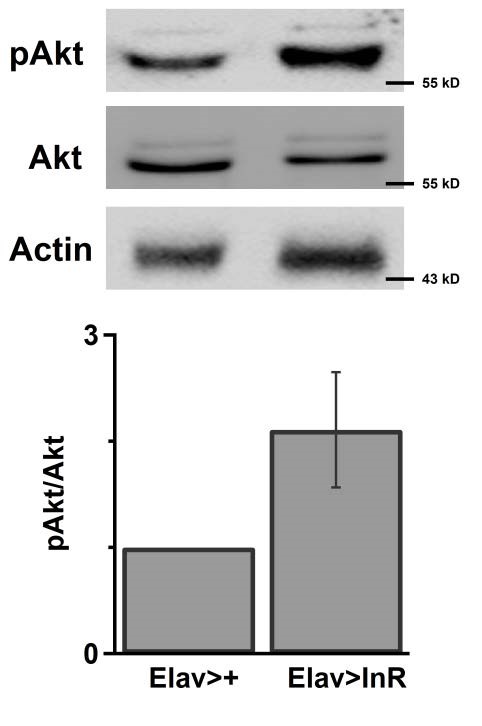


Fig. S3. Neuronal expression of InR increases Akt phosphorylation. The brains of 14 dae Elav>InR^wt^ flies were used to do Western blot assay. N=12. p<0.06 between

Elav>+ and Elav>InR^wt^


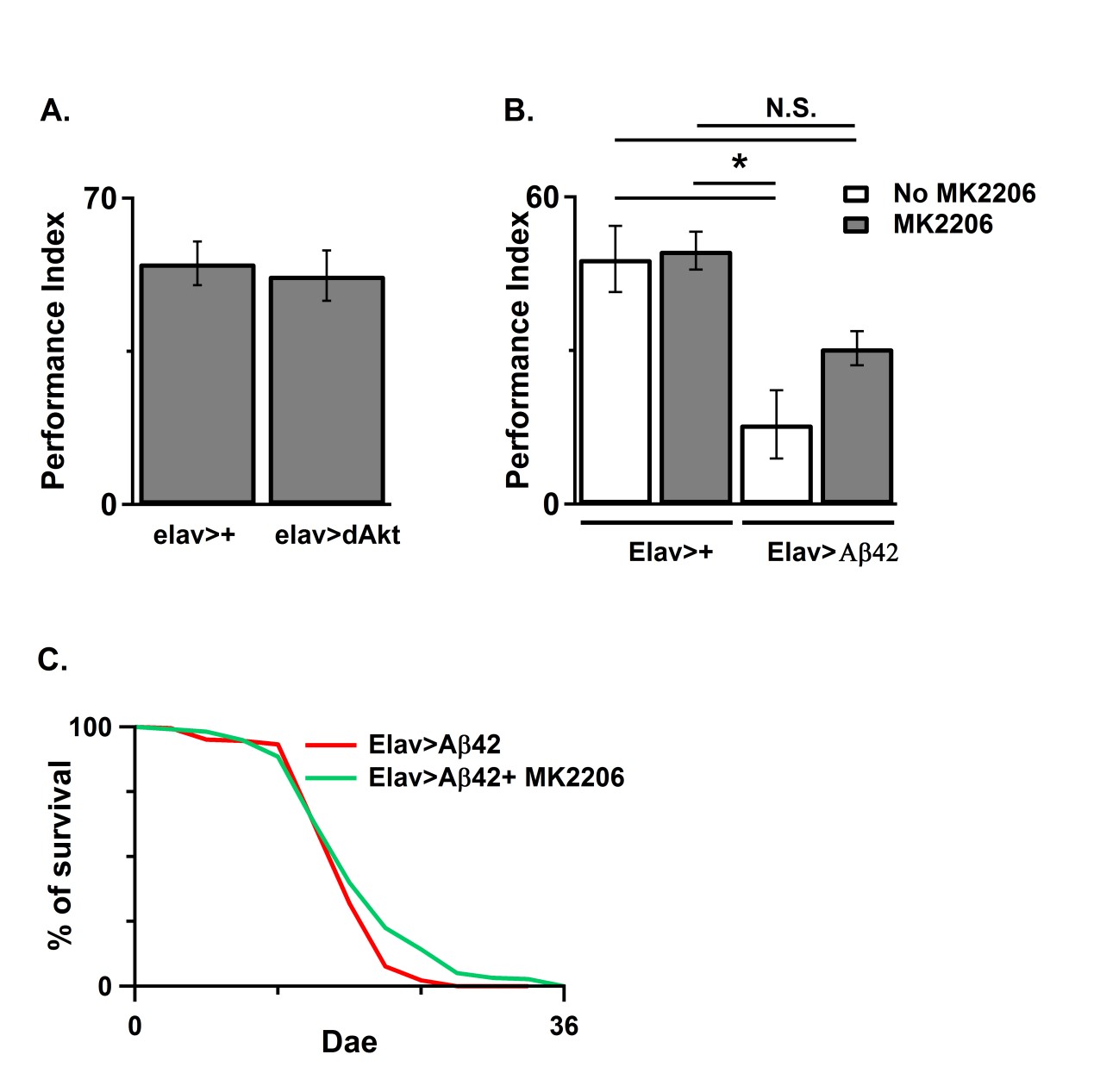


Fig. S4. Akt inhibitor reversed A_β_42-induced learning deficit. A. There was no observed learning deficit in the 5 days old Akt flies. B. Aβ42 flies treated with 100µM MK2206 for 7-days showed a better learning performance. N=8-10. *p<0.05. C.

Aβ42 treated with 100µM MK2206 showed delayed early death compared to the A_β_42 flies without treatment. The delay was become obvious in the later stage, after 20 days. n=210-220. p<0.0001 log-rank test.
